# Small non-coding RNAs transfer through mammalian placenta and directly regulate fetal gene expression

**DOI:** 10.1007/s13238-015-0156-2

**Published:** 2015-05-12

**Authors:** Jing Li, Yujing Zhang, Dameng Li, Yuchen Liu, Danping Chu, Xiaohong Jiang, Dongxia Hou, Ke Zen, Chen-Yu Zhang

**Affiliations:** State Key Laboratory of Pharmaceutical Biotechnology, Nanjing Advanced Institute for Life Sciences (NAILS), Nanjing University School of Life Sciences, Jiangsu Engineering Research Center for MicroRNA Biology and Biotechnology, Nanjing University, 22 Hankou Road, Nanjing, 210093 Jiangsu China

**Table:** 

SMALL NON-CODING RNA CAN TRANSFER THROUGH THE PLACENTA AND REGULATE FETAL DEVELOPMENT	
Increasing evidence demonstrated that exogenous plant miRNAs in the diet can be absorbed and are found in mammalian tissues. These miRNAs influence the physiological and pathological progression of the recipient. In this issue, Jing Li and colleagues further reveal that exogenous/endogenous small non-coding RNA in the maternal system can transfer through the placenta to the fetal side and influence fetal development and health. These surprising finding demonstrate that maternal miRNAs profiling are very important to fetal development, which means maternal diet and the healthy status may influence fetal development and the postnatal morbidity via transplacental small non-coding RNAs. Furthermore, this finding also proposes a brand new potential strategy to treat fetal diseases in utero. Combining with gene therapy, maternal administrating represents a new trend for treating fetal diseases. *—Yali Hiu* 1	

The placenta is a vital organ on which the survival and growth of the fetus are critically dependent. It forms the interface between the maternal and fetal environments, facilitates the exchange of gases, nutrients, and waste products between the mother and baby and also acts as a barrier against the maternal immune system (Watson and Cross, 2005). In humans and rodents, the overall structures and the molecular mechanisms of placental development are thought to be quite similar; thus, the mouse is commonly used as a model to study the placenta. In mice, placental development begins at embryonic day (E) 3.5, and the placenta is considered mature at E 14.5 (Rossant and Cross, 2001). Nutrients are transferred through the placenta via three mechanisms: passive diffusion, facilitated diffusion, and active transport (van der Aa et al., 1998).

MiRNAs are steadily present in the circulatory system and can serve as biomarkers for cancers and other diseases (Chen et al., 2008). These plasma miRNAs are actively secreted from tissues and are mainly packaged by microvesicles (MVs), which will then deliver these miRNAs and facilitate their entry into distance recipient tissues to regulate gene expression (Zhang et al., 2010). Further study revealed that exogenous plant MIR168 in food can be absorbed by the mammalian small intestine and is present in animal plasma. Exogenous plasma MIR168 then enters the liver and regulates the target gene low-density lipoprotein (LDL) receptor adapter protein 1 (LDLRAP1), showing that exogenous plant miRNAs play an important role in regulating physiological and pathological progression (Zhang et al., 2012).

In the present study, we were surprised to find exogenous plant miRNAs in human umbilical cord blood and amniotic fluid. Thus, based upon these findings, we propose the hypothesis that endogenous/exogenous small non-coding RNAs, including miRNAs and siRNAs, can transfer through the placenta and enter the fetus to regulate fetal development.

Upon detecting the global miRNA expression profile in the umbilical cord blood and amniotic fluid of healthy Chinese pregnant women, we found that exogenous plant miRNAs were consistently present in human umbilical cord blood and amniotic fluid, which belongs to the fetal circulation system (Fig. 1A and Table S1A). The presence of plant miRNAs in fetal umbilical cord blood and amniotic fluid suggests that exogenous miRNAs could transfer through the placenta to the fetal side. Plant miRNAs were also found in the mouse fetuses by quantitative RT-PCR (Fig. 1B). Taken together, the above results suggest that miRNA may transfer through the placenta to the fetal side.

**Figure 1:**
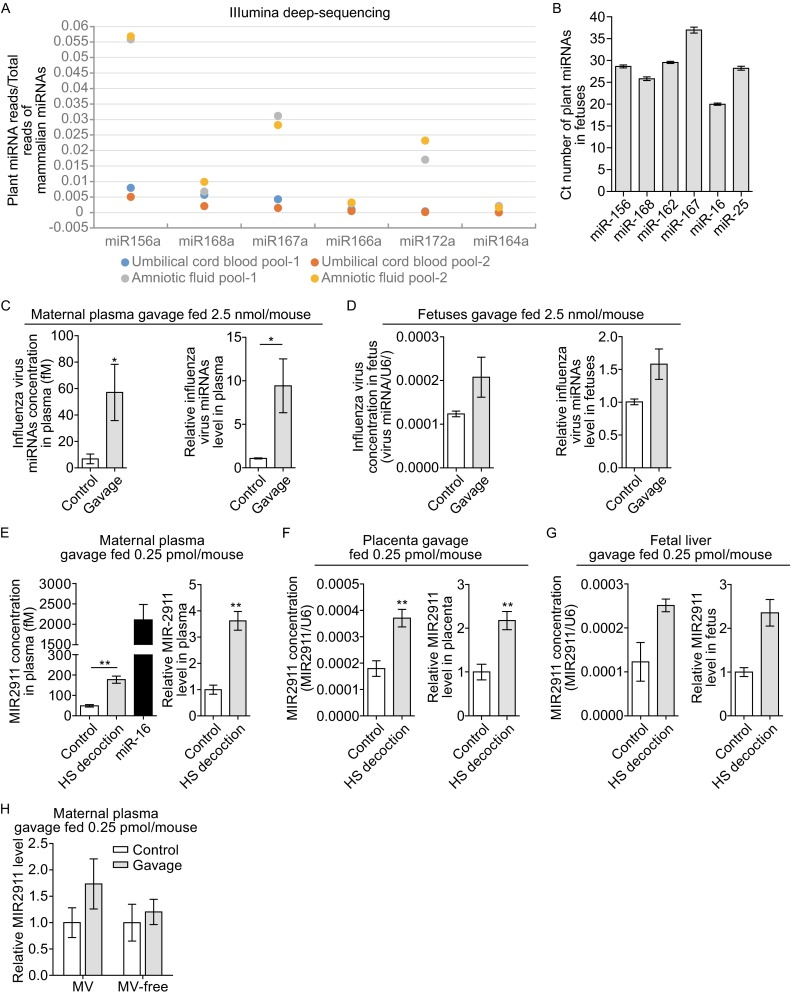
**Small non-coding RNAs can transfer through placenta and enter into fetuses**. (A) The levels (sequencing reads) of 6 plant miRNAs detected by Illumina deep sequencing in umbilical cord blood and amniotic fluid from healthy pregnant women. (B) The Ct number of plant miRNAs in mouse fetuses. (C) The concentrations and relative levels of exogenous synthetic influenza virus miRNAs in maternal plasma. (D) The concentrations and relative levels of exogenous synthetic influenza virus miRNAs in fetuses. (E) The concentrations and relative levels of miR2911 in maternal plasma. (F) The concentrations and relative levels of miR2911 in placentas. (G) The concentrations and relative levels of miR2911 in fetal livers. (H) The levels of MIR2911 in maternal plasma. Each group *n* = 5, and all control groups were treated with the equal volume saline

To further investigate whether mature miRNAs can pass through the placenta, an exogenous mature miRNA originated from the influenza virus was employed to trace the miRNA in mice. We synthesised mature miRNAs of the influenza virus and gavage fed these miRNAs to pregnant mice that had been pregnant for at least 14 days and had mature placenta. The level of the influenza virus miRNAs in maternal plasma were elevated significantly 3 h after gavage feeding (Fig. 1C). In the fetal liver, we also observed an increase in the exogenous miRNA level (Fig. 1D).

Next, we sought to further investigate whether mature miRNAs in plants can pass through the placenta and efficiently enter fetal organs. Honeysuckle (HS) is a famous traditional Chinese herb. It was chosen because of the specific enrichment of MIR2911. Thus, MIR2911 can serve as a tool to trace the transplacental transmission of exogenous plant miRNAs. According to our previous study, MIR2911 is hardly degraded during boiling, so it is stably present in the HS decoction (Zhou et al., 2015). The mice were gavage fed with 0.5 mL HS decoction, and the maternal plasma and fetal liver were collected 3 h after gavage feeding. Precautions were taken to prevent contamination of the fetus with the maternal sample. The results revealed that the MIR2911 levels were elevated ~3.5-fold in the maternal plasma, reaching 180 fM (Fig. 1E). The MIR2911 level also increased in the placenta (Fig. 1F). Interestingly, MIR2911 was significantly elevated (~2.5-fold) in the fetuses (Fig. 1G), suggesting that plant MIR2911 in HS can efficiently transfer through the placenta to enter the fetal liver. Thus, plant miRNAs in the diet can pass not only through mouse GI tract to enter the maternal plasma but also through the placenta to enter into the fetus, thereby influencing growth and development. We next measured the amount of circulating MIR2911 in MVs compared to that in MV-free plasma. After gavage feeding, MIR2911 in MV-free plasma did not vary; in contrast, MIR2911 was increased in MVs (Fig. 1H), suggesting that circulating MIR2911 was primarily present in MVs and that the transplacental transmission may involve an MV-mediated pathway.

We then sought to explore whether double-stranded small interfering RNA (siRNA) can transfer through the placenta. First, a fluorescently labelled siRNA was utilised to trace the transplacental transmission of siRNA. The results showed that the fluorescent signal can be detected by confocal microscopy in the fetal liver, suggesting that siRNA can pass through the placenta and enter the fetal liver (Fig. 2A). Alpha-fetoprotein (AFP) is a type of major plasma protein produced in the yolk sac and the liver during fetal development. The plasma level of AFP decreases rapidly after birth; thus, AFP can be considered a protein that is specifically expressed during fetal development. We then performed further experiments to demonstrate that exogenous miRNAs in the fetus have specific biological properties. A synthetic siRNA targeting AFP mRNA was synthesised, and the interference efficiency was verified in a mouse hepatocellular carcinoma cell line (Hepa 1–6) (Fig. S1A). The mice were gavage fed with the siRNAs at 2 nmol and 5 nmol, respectively. After 3 h, the siRNA levels were significantly elevated in the maternal plasma and fetal liver in the 5 nmol group (Fig. 2B and 2C). Interestingly, the mRNA and protein level of AFP was dramatically down-regulated in mice treated with 5 nmol siRNAs, suggesting that exogenous siRNAs delivered from the mother to the fetus by transplacental transmission, could regulate fetal gene expression (Fig. 2D and 2E). Given that transplacental transmission may involve an MV-mediated pathway, we directly injected MVs loaded with siRNAs. The results revealed that siRNA levels increased 2-fold in the fetus (Fig. 2F) and that AFP mRNA levels were significantly decreased (Fig. 2G). Taken together, these results suggested that siRNAs can be delivered through the placenta and that siRNAs have biological functions in the fetus.

**Figure 2:**
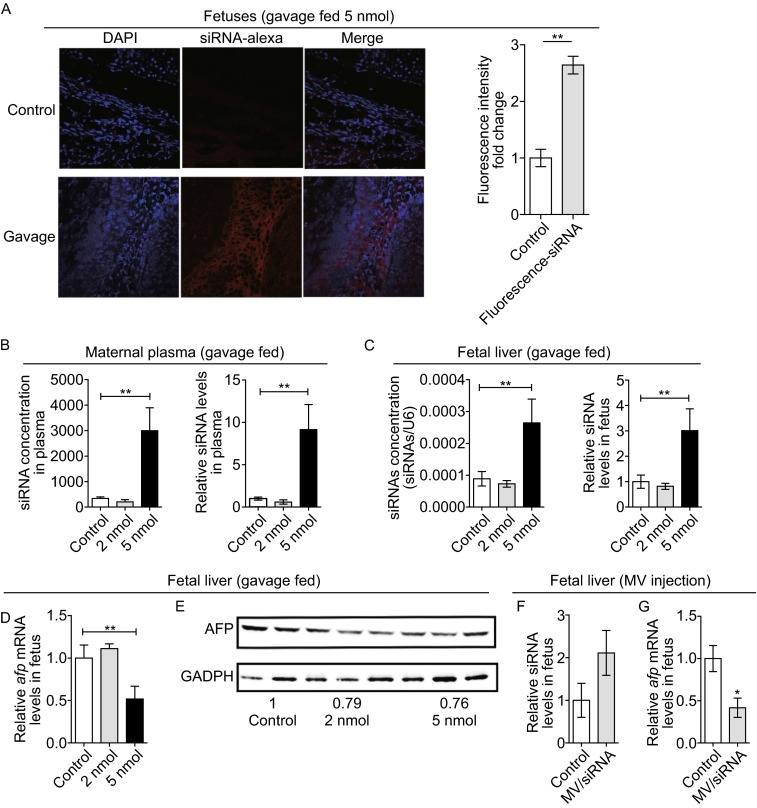
**The synthetic siRNA can pass through placenta and regulate gene expression in the fetal liver**. (A) The confocal microscopy photos of fetal liver after gavage fed with saline or fluorescence-labeled siRNA. (B) The concentrations and relative levels of siRNA in maternal plasma. (C) The concentrations and relative levels of siRNA in fetal livers. (D) The mRNA level of *afp* in fetal livers. (E) The protein level of AFP in fetal livers. (F) The relative levels of siRNA in fetal liver after administration of siRNA by intravenous injection. (G) The mRNA levels of *afp* in fetal livers after intravenous injection. Each group *n* = 5, and all control groups were treated with the equal volume saline

The placenta is key to mammalian fetal development. Many endogenous compounds can transfer through the placenta. In addition to these compounds that are necessary for fetal development, cells, viruses, and DNA can also pass through the placenta. MiRNAs are a class of tiny molecules with expansive functions in many pathological and physiological process (Bartel, 2004). Because secreted miRNAs packaged by MVs are stably present in the circulatory system and are simultaneously transported to distant recipient cells by MVs (Zhang et al., 2010), we believe that miRNAs have great potential to be transferred through the placenta by an MV-mediated mechanism. Given that exogenous plant miRNAs can be absorbed by the GI tract and can regulate gene expression across kingdoms (Zhang et al., 2012; Zhou et al., 2015), exogenous plant miRNAs are believed to be absorbed by the mother and transferred into the fetus.

This is the first study to examine the transplacental transmission of small-noncoding RNAs. The results revealed that both single- and double-strand of small non-coding RNAs can transfer through the placenta. Plant miRNAs are the major source of exogenous RNAs for humans. Thus, it is important to reveal whether natural miRNAs in plants can pass through the placenta and regulate fetal development. Our results revealed that MIR2911 from honeysuckle can be absorbed and delivered into the fetal liver, suggesting that not only the proteins, carbohydrates, and aliphatic acids but also the nucleic acids in the maternal diet can be taken into the fetus. It is mentionable that the majority of increased MIR2911 was present in MVs, supporting that the transplacental transmission of miRNAs is mediated by the MV pathway. Then, functional siRNAs for AFP were employed to identify the effect of non-coding RNAs in the fetus; strikingly, siRNAs ingested by maternal mice dramatically reduced the AFP expression level. AFP is a critical protein in fetal development. It has been demonstrated that AFP plays a role in neuronal development because it serves as the binding protein for arachidonic acid (AA) and docosahexaenoic acid (DHA), which are important for neuronal development (Mizejewski, 2004). Our findings demonstrate that mother-derived miRNAs participate in fetal epigenetic regulation during pregnancy, which may supply a new explanation for some epigenetic phenomenon and congenital diseases.

Conventional wisdom holds that essential nutrients for fetal development are carbohydrates, fats, dietary fibre, minerals, protein, vitamins, and water. Now, miRNAs should be another nutrient. Given that maternal-fetal transplacental miRNAs play an important role in fetal development, miRNA profiling of the maternal diet is a new parameter that requires attention. The dietary patterns of the mother will influence the fetus or even determine the postnatal health status. Dietary bias or other unhealthy dietary habits would also affect the fetal health by disrupting the balance of transplacental miRNAs. Along the same lines, the pathological status of the mother will result an abnormal endogenous miRNA profile per se, which will also influence fetal health. Our findings also allow for the proposal of a brand new strategy for disease treatment in utero. According to previous knowledge, various common viruses such as human immunodeficiency virus (HIV), Rubella virus, human papillomavirus (HPV) and hepatitis C can be vertically transmitted from the mother to the fetus (Atreya et al., 2004; Castellsague et al., 2009; Le Campion et al., 2012; Soilleux et al., 2001). The methods to treat these diseases remain limited, and the maternal exposure to medicine may trigger fetal DNA damage or may affect mitochondrial DNA (mtDNA) (Brogly et al., 2011). Previous study demonstrated that microvesicles-mediated gene therapy is an ideal strategy to treat various diseases (Li et al., 2013; Liu et al., 2013; van den Boorn et al., 2011; Zhang et al., 2014). Our previous work also reported that MIR2911, found in Chinese herbs, can be absorbed and accumulated in the lungs to inhibit the influenza A virus (Zhou et al., 2015). Thus, gene therapy by administrating virus-targeted artificial siRNA to the mother represents a new trend for treat viruses in utero. The virus-targeted siRNA not only treats the mother but also inhibits the virus from vertically transmitting to the fetus.

In conclusion, the present study provides the first evidence that small non-coding RNA can be transplacentally transmitted from the mother to the fetus. Although the data suggest that transplacental transmission may be mediated by MVs, further study must be performed to uncover the mechanism of the transplacental transmission of small non-coding RNA. Our data suggest another important parameter for fetal development and health and a new potential disease treatment in utero.

## Electronic supplementary material

Supplementary material 1 (PDF 113 kb)

## Footnotes

1 Department of Obstetrics and Gynecology, Nanjing Drum Tower Hospital, Nanjing University Medical School.
