# Supplementary material for: Small non-coding RNAs transfer through mammalian placenta and directly regulate fetal gene expression
Source: Protein Cell. 2015 May 12;6(6):391–6. doi: 10.1007/s13238-015-0156-2 (PMC4444809; doi:10.1007/s13238-015-0156-2)
Supplement: Supplementary file 1 — Supplementary material 1 (PDF 113 kb) [file 13238_2015_156_MOESM1_ESM.pdf]

Supplementary information

Materials and Methods

Reagents, cells and antibodies

Synthetic RNA molecules, including mature miRNA oligonucleotides of the influenza virus, fluorescently labelled Alexa Fluor® 555 siRNA and AFP siRNA were purchased from Invitrogen (Carlsbad, CA, USA). The sequence of the mature miRNA oligonucleotides of the influenza virus were as follows: 5'-GAACCUGGCGAUCCGAAUGCAU-3'. The mouse hepatocellular carcinoma cell line Hepa1-6 was purchased from the Institute of Biochemistry and Cell Biology, Shanghai Institutes for Biological Sciences, Chinese Academy of Sciences (Shanghai, China). Hepa1-6 cells were maintained at 37°C in a humidified 5% CO_2 incubator with Dulbecco's modified Eagle's medium (Gibco, CA, USA) containing 10% fetal bovine plasma (FBS, Gibco), 100 units/ml of penicillin, and 100 μg/ml of streptomycin. Alpha-fetoprotein and GADPH antibodies were purchased from Santa Cruz (Santa Cruz, CA, USA).

Illumina deep sequencing

Small RNAs were isolated from umbilical cord blood and amniotic fluid donated by healthy, pregnant Chinese women. All of the human samples were collected from Illumina, and the sequencing of RNA samples was performed by BGI (Shenzhen, China). After removing the adaptor sequences from the raw data, the clean reads were compared to known miRNA precursors and mature miRNAs from miRBase database 14.0 to identify conserved plant miRNAs based on the Smith-Waterman algorithm.

Honeysuckle (HS) decoction preparation

The traditional Chinese medicine honeysuckle was purchased from a Chinese herbal medicine shop. First, 5 g honeysuckle was boiled with 200 ml tri-distilled water for 30 min, and the decoction was then concentrated to 5 ml (final concentration: 1 g HS/ml decoction). the HS decoction was prepared by boiling 5 g HS in 200 ml double-distilled water for 30 min; the decoction was then concentrated to 3 ml (final concentration: 1.67 g HS/ml decoction). According to the previous study, the MIR2911 concentration in 0.2 g HS/ml decoction is 0.06 pmol/ml, thus, the MIR2911 concentration in a decoction of 1.67 g HS/ml is equal to 0.5 pmol/ml. Each mouse was gavage fed with 0.5 ml decoction, which was equivalent to 0.5 g honeysuckle for each mouse.

Exosome isolation

Exosomes were isolated from plasma by using the Total Exosomes Isolation Kit (4484450, Invitrogen) according to the manufacturer's instruction.

RNA isolation and RT-qPCR of mature miRNAs

Total RNA was extracted from the tissues using TRIzol Reagent (Invitrogen) according to the manufacturer's instruction. Small RNAs from plasma were extracted using the MicroRNA Kit (BioTeKe, Beijing, China). Quantitative RT-PCR was performed using TaqMan miRNA probes (Applied Biosystems, Foster City, CA, USA) and a Roche PCR machine (Roche, Basel,

Switzerland) according to the manufacturer's instructions. After the reaction, the C_T values were determined using the fixed threshold setting. The expression level of miRNAs in tissues was normalised to the U6 snRNA level using the $2^{-\Delta\Delta Ct}$ method. To calculate the absolute expression levels of the target miRNAs, a series of synthetic miRNA oligonucleotides at known concentrations were reverse transcribed and amplified. The absolute amount of each miRNA was then calculated in reference to the standard curve.

Cryosectioning and confocal microscopy analysis of fluorescently labelled siRNAs

Whole fetal mice were collected from mothers that had been previously gavage fed with fluorescently labelled siRNA. Then, the fetal mice embedded in OTC were flash frozen and sectioned on a freezing microtome. After washing in PBS three times (3 minutes for each), the sections were treated with paraformaldehyde at room temperature for 30 minutes. After washing, the sections were incubated with antifade reagent (P36931, Invitrogen) and were then observed by confocal microscopy (FV1000; Olympus, Tokyo). The excitation wavelength was 405 nm for DAPI and 532 nm for fluorescently labelled siRNA. The image size was 1024 × 1024 pixels.

Animal studies

All animal experimental procedures were carried out in accordance with the National Institutes of Health Guide for the Care and Use of Laboratory Animals and were approved by the Animal Care Committee of Nanjing University (Nanjing, China). Eight-week-old female C57BL/6J mice were maintained on a 12 h-light/dark cycle in a pathogen-free animal facility at Nanjing University and were mated with male mice of the same age. Eighteen days after mating, when the placenta matured, the mother mice were gavage fed with various exogenous small RNAs, and then the maternal plasma and the fetal liver were collected after 3 hr. Precautions were taken to prevent contamination of the fetus with the maternal sample.

To study whether the single-stranded mature miRNAs could pass through the placenta, the mother mice were gavage fed with 2.5 nmol synthesised influenza virus miRNA, while the control group were treated with saline at the same volume.

To identify the transplacental transmission of natural exogenous plant miRNAs, the mother mice were gavage-fed with 0.5 ml HS decoction (MIR2911 concentration: 0.5 nM). The control group was treated with same volume of water.

To identify the transplacental transmission of double-stranded siRNAs, the mother mice were gavage fed with 2 nmol or 5 nmol AFP siRNA. The control group was treated with saline.

Supplementary figures and tables

Supplementary Table 1. The level of plant miRNAs in human cord blood and amniotic fluid.

Name	ref miRNA	umbilical cord blood pool-1	umbilical cord blood pool-2	amniotic fluid pool-1	amniotic fluid pool-2
MIR156a	osa-miR156a	2305	3023	396	867
MIR156b	osa-miR156b	2334	3087	396	867
MIR156c	osa-miR156c	2334	3085	396	867
MIR156d	osa-miR156d	2637	3371	427	975
MIR156e	osa-miR156e	2305	3022	396	867
MIR156f	osa-miR156f	2632	3371	427	975
MIR156g	osa-miR156g	2334	3085	396	867
MIR156h	osa-miR156h	2632	3371	427	975
MIR156i	osa-miR156i	2305	3022	396	867
MIR156j	osa-miR156j	2632	3371	427	975
MIR156k	osa-miR156k	53	105	97	309
MIR164a	osa-miR164a	30	36	15	25
MIR164b	osa-miR164b	30	36	15	25
MIR164d	osa-miR164d	30	36	15	25
MIR164e	osa-miR164e	30	36	15	25
MIR164f	osa-miR164f	30	36	15	25
MIR166a	osa-miR166a	282	295	21	49
MIR166b	osa-miR166b	282	295	21	49
MIR166c	osa-miR166c	290	297	21	23
MIR166d	osa-miR166d	282	295	21	49
MIR166f	osa-miR166f	282	295	21	49
MIR166g	osa-miR166g	282	295	21	48
MIR166h	osa-miR166h	282	295	21	22
MIR166k	osa-miR166k	15	0	0	0
MIR166l	osa-miR166l	15	0	0	0
MIR166m	osa-miR166m	282	295	21	22
MIR166n	osa-miR166n	290	297	21	23
MIR167a	osa-miR167a	1241	875	221	430
MIR167b	osa-miR167b	1241	875	221	430
MIR167c	osa-miR167c	1241	875	221	430
MIR167d	osa-miR167d	1267	915	221	430
MIR167e	osa-miR167e	1267	910	221	430
MIR167f	osa-miR167f	1267	910	221	430
MIR167g	osa-miR167g	1267	910	221	430
MIR167h	osa-miR167h	1267	910	221	430
MIR167i	osa-miR167i	1267	915	221	430
MIR167j	osa-miR167j	1267	915	221	430
MIR168a	osa-miR168a	1645	1243	48	151

Name	ref miRNA	umbilical cord blood pool-1	umbilical cord blood pool-2	amniotic fluid pool-1	amniotic fluid pool-2
MIR172a	osa-miR172a	110	72	121	354
MIR172d	osa-miR172d	110	72	121	354
MIR393	osa-miR393	26	16	0	0
MIR393b	osa-miR393b	26	18	0	0
MIR396d	osa-miR396d	10	8	0	0
MIR396e	osa-miR396e	10	8	0	0
MIR396f	osa-miR396f	10	8	0	0
MIR396g	osa-miR396g	10	8	0	0
MIR396h	osa-miR396h	10	8	0	0
MIR396i	osa-miR396i	10	8	0	0
MIR397a	osa-miR397a	11	3	0	0
MIR528	osa-miR528	26	29	8	12
MIR535	osa-miR535	23	20	6	3
MIR1318	osa-miR1318	55	45	0	0
MIR1432	osa-miR1432	55	45	0	0

Supplementary figure 1. AFP siRNA influence efficiency.

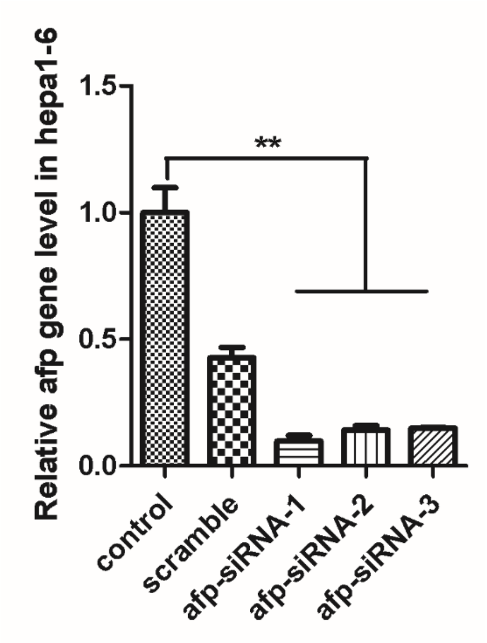

Supplementary figure 1. The relative level of *afp* mRNA after the cell untreated or treated with scramble RNA or siRNA
